# Panda Unit, a Mother-Baby Unit Nested in a Neonatal Care Service

**DOI:** 10.3389/fpsyt.2022.889557

**Published:** 2022-08-09

**Authors:** Lisa Vitte, Cyriaque Hauguel, Vincent Benoit, Marie-Camille Genet, Jessica Letot, Henri Bruel, Florian Delaunay, Pascal Le Roux, Priscille Gerardin, Emmanuel Devouche, Gisèle Apter

**Affiliations:** ^1^Hospital Group Du Havre, Le Havre, France; ^2^Laboratoire de Psychopathologie et Processus de Santé, Université Paris Descartes, Paris, France; ^3^Service Universitaire de Pédopsychiatrie, Hospital Group Du Havre, Le Havre, France; ^4^Chef de Service Néonatologie, Hospital Group Du Havre, Le Havre, France; ^5^Chef de Pôle Maternité, Hospital Group Du Havre, Le Havre, France; ^6^Chef de Pôle Pédiatrie, Hospital Group Du Havre, Le Havre, France; ^7^Faculté de Médecine et de Pharmacie, Université de Rouen, Mont-Saint-Aignan, France

**Keywords:** mother-baby unit, neonatal care service, perinatal psychiatry, early perinatal care, interactive dysregulation

## Abstract

The PANDA unit is a full-time mother-baby hospitalization unit based on an original model of care for vulnerable dyads. It is located within a neonatal unit allowing tripartite care (perinatal psychiatry, neonatology and post-natal care). It thus differs from traditional mother-baby units in its close links with the other perinatal care actors, allowing comprehensive health and mental health care in the immediate post-partum period. Patients admitted to the Panda Unit may have been referred during the antenatal period or taken into care in an emergency if the mother's clinical condition requires it, in the aftermath of childbirth. During their stay, the dyads are evaluated daily by a perinatal psychiatrist. This includes assessment of maternal clinical state, the newborn's development and the quality of mother-infant interactions. During the first 6 months of use, 24 dyads have benefited from PANDA care. Three women among 5 were admitted during the antenatal period and almost one-third were aged under 21. The first primary diagnosis during the antepartum was major depressive disorder, two-fold that of personality disorder or bipolar disorder alone. At the end of PANDA stay, close to 3 women among 4 were back to their home with their child, and an out-of-home placement was mandated for 4 infants. PANDA unit is a step toward continuous and comprehensive integrative care. The mother and baby do not leave the maternity ward, and management of mother, baby, and their interactions can start immediately after birth. Considering the importance of the first months of life in the establishment of fundamental links and bonding, PANDA offers an innovative opportunity for what we hope will be both therapeutic and preventive for at-risk dyads. The detection, and ultimately prevention and management of risk of abuse and neglect is another major challenge that this unit hopes to address from the very beginning.

## Introduction

The perinatal period is now recognized as a one of increased vulnerability for the mental health of women and their offspring. Indeed, nearly 20% of mothers are concerned by the novel or reiterated occurrence of a psychiatric disorder during this time (1). The psychiatric disorders found, include a wide range of disorders: perinatal depressive and anxiety disorders, psychotic disorders, bipolar disorders often associated with personality disorders including Borderline Personality Disorder (2–4). Severe disorders are not rare, 2–3% of births are subject to major depressive episodes and close to 10% have after-effects of possibly associated traumas often linked to history of abuse and/or neglect (5–7). The 6th report of the National Confidential Enquiry on Maternal Deaths published in France in 2021 thus places suicide as one of the two leading causes of maternal mortality in the first year of life of the baby in a close tie with cardio-vascular diseases (8). These results are in line with American and UK studies showing the same two leading causes of death during pregnancy and up to 6 weeks postpartum, while after 6 weeks postpartum, suicide becomes the leading cause until 1 year postpartum.

Conversely, for offspring, *in utero* development is a highly sensitive period. Many endocrine and toxicological factors are known to have a negative influence on neurodevelopment (9, 10). More recently, the impact of history of maternal childhood maltreatment has shown variations in newborn brain structure (11). Maternal borderline disorders overlap with history of trauma and abuse. And personality disorders have been identified as the leading cause of placement in foster care among patients in mother-baby units in France (12).

The early onset of interactive dysfunctions observed in studies underlines specific periods of sensitivity that extend beyond pregnancy and into the first weeks or months of the infant's life. Recent studies on the communication skills of newborns in non-risk dyads highlight an ability to engage in a conversational exchange with their mother (13) or father (14). They include a capacity to initiate or re-initiate an exchange with a foreign partner (15, 16). Buil et al. (17) also observe the existence of multiple interactive exchanges during skin-to-skin contact in babies born very prematurely from 18 days of life. This is achievable when optimal skin-to-skin positioning makes it possible for mothers to have face to face contact with their newborn. Ultimately, we know that as early as 2–3 months of age, the infant of a mother diagnosed with borderline personality disorder/disorganized attachment is already engaged in a dysfunctional interactive rhythmicity, therefore already headed toward an at-risk developmental trajectory (18–20). As Apter et al. (3) highlight in their literature review, findings converge to reveal that offspring of parents (generally mothers) with psychiatric disorders present a higher risk for all mental disorders during childhood and adolescence. A wide range of disorders have been repeatdly found in children, adolescent, and finally adult offspring of mothers with mood and anxiety disorders (21, 22).

Whether it is a question of care for the mother, the baby or that of communication within the dyad, the studies mentioned plead for the earliest possible perinatal care, ideally from before birth.

The public health issues concerning mother-baby dyads have thus pushed to rethink care during the perinatal period both in terms of human resources and appropriate and specific care arrangements. Mother-Baby Hospitalization Units (MBU) are tertiary care facilities and have been found an effective means of caring for vulnerable dyads in France and other European Countries (23, 24). Indeed, as pointed out by Griffith et al. (24), findings suggest that specialist perinatal inpatient care is considered preferable to generic care in the perinatal period from both service user and staff perspectives. However, although MBU enabled severely ill women to remain with their infants during treatment (25, 26) remind that the provision of psychiatric inpatient MBUs around the world varies considerably and so the benefits for both the mother and the baby.

Increased collaboration between perinatal and non-perinatal services could help improve perinatal expertise on general psychiatric wards, while further expansion of perinatal services (e.g., to cater for women currently considered too high risk for MBUs and for those discharged from inpatient settings) could tackle other shortfalls in care.

In France, MBU units have diverse settings due to the heterogeneity of the way they have been set up since the 1980's. They can be located in a child or adult psychiatry department, in a psychiatric or general hospital. Some MBUs allow fathers to be accommodated, while others are only day-care settings (27). Since the creation of the first MBU in France in 1980 in Créteil (28), several MBUs have emerged: in April 2020, the French-speaking Marcé site^1^ counted 15 full-time MBUs, and 14 day-hospital MBUs. Whatever their form or capacity (number of “beds”), mother-baby units are characterized by the fact that they theoretically offer joint care for the mother and newborn, adding treatment of current psychiatric maternal disorder to prevention and management of its impact on infant health and mental health. As Nezelof et al. [(29), p. 368] point out, “It is not only a matter of providing care for a mother in psychological distress, but also of fostering the development of maternal feeling and function, of being attentive to the child's development and of supporting the bonds that unite them.” It is a matter of thinking in twos and threes (30).

Despite the awareness of the needs in the field, existing practices are pointed out as heterogeneous, and do not sufficiently cover the whole population (31). In 2017, Brockington et al. (32) pointed out that in France, the supply of mother-baby hospitalization beds met half of the population needs, a finding that also affected England, New Zealand and Switzerland. In September 2020, the report of the “French Commission on the first 1,000 days^2^” emphasized that “specialized follow-up structures exist (mother-child part-time hospitalization, outpatient units and specialized consultations), however, that it was extremely difficult to compare the way they operate. They do not have the same names, they are very unevenly distributed throughout the country, they have heterogeneous sources of funding, and the overall supply falls far short of needs.” Following this report, the Secretary of State for Child Protection announced an investment to develop 10 new MBUs. Thus, in 2021, several additional MBUs were to be funded. This increase in the supply of beds, with a view to a balanced distribution over the national territory is a welcome development. Furthermore, as Glangeaud-Freudenthal et al. (33) pointed out in 2014, MBUs need to be part of a health and perinatal health networks that include maternity wards, neonatal care, and community resources. In other words, intervention is needed before birth, during birth and after birth in a multidisciplinary setting. There can be no perinatal health without perinatal mental health and reciprocally.

In its current form, most UMB's do not allow for this very early, dual preventive and curative care within the maternity ward, and/or when the newborn baby requires immediate specific pediatric care and/or the mother emergency psychiatric care. In order to fill this gap, with this dual immediate peripartum approach, two full-time mother-baby hospitalization beds were opened at the Groupe Hospitalier du Havre in April 2021 within the maternity ward and neonatal “Kangaroo Unit”. These nested beds, coined “PANDA” offer an innovative model of care, with an integrated economic model (both medical and psychiatric funds). The aim of this article is to describe this Panda model of care and to present the first data available 6 months after the inclusion of the first dyads.

## Methods and Results

### The PANDA Unit

The PANDA Unit was born out of a growing need to care for mothers with mental health problems in Normandy maternity wards. There was up until 2021 no joint mother-infant hospitalization in the whole of the Normandy region (30,000 square kilometers and 3.4 Million population). PANDA consists of two experimental full-time mother-baby hospitalization beds within the Kangaroo Unit, i.e., nested in the pediatric unit of one of the three major tertiary care hospitals of the region Le Havre, situated at close to equal distance of the other two.

The PANDA unit is composed of a multidisciplinary team and is included in the Infant and Child Psychiatry Department. It includes two perinatal psychiatrists (perinatal psychiatrists are in France trained both in child and adult perinatal psychiatry), a mental health professional with knowledge of special needs and severe mental health disorders and a nursery nurse (all part-time). Pediatric care is provided simultaneously with the PANDA hospitalization, *via* the pediatricians and the nursing team of the Kangaroo Unit. The same applies to obstetrical care following the birth of a child *via* the midwives working in the pathological pregnancies' unit. The medical team works 7 days a week. One of the carers is always on site 6/7 days a week. In addition to the daily transmissions, clinical situations are discussed on a weekly basis within Panda Staff and both with Obstetric and Neonatal staff.

Whenever possible, patients admitted to the Panda Unit are first seen by the perinatal psychiatrist during a pre-admission interview during the antenatal period. However, they may be taken into care in an emergency procedure, if the mother's clinical condition requires it in the aftermath of childbirth. Motives for hospitalization in the PANDA Unit concern both maternal psychiatric disorders, predictable (history) major risk of difficulties in mother-baby interactions, and potential negative impact of maternal pathology on newborn (birthweight, prematurity, and/or withdrawal of substances and medication). Panda care is focused on both maternal illness and its consequences on the newborn and infant, whereas the Kangaroo unit is specifically part of the pediatric neonatal ward, therefore referral to Kangaroo is linked only to infant developmental risk and illness. There is no organized care for the accompanying adult, i.e., the mother.

As in an MBU, PANDA Unit offers joint care for the mother and baby and scaffolds the development of an ameliorated qualitative mother-infant communication. What comes together here is the management of a spectrum of maternal psychiatric disorders that is expected to be wider than usually, with unforeseen referrals due to a closely related ObGyn and neonatal intertwined services and a closely knitted regional network (https://www.reseaux-perinat-hn.com/).

### PANDA Hospitalization

During the hospitalization, the dyads are evaluated daily by the perinatal psychiatrist who assesses the maternal clinical evolution, the newborn's development and the quality of mother-baby interactions.

### Maternal Disorders

Maternal clinical assessment is based on semi-structured perinatal psychiatric interviews conducted in pairs with a mental health care worker (nursery nurse or educator) from the PANDA unit, and on scales or tools that assess the childbirth experience, maternal depression and anxiety, or childbirth disorders.

Situations of risk or of proven abuse, whether during childhood or still ongoing are also assessed by recognizing the risk factors for abuse documented by the National Health Agency (HAS, Haute Autorité de Santé), identifying notes of concern and reports during the perinatal period, and using the Steinhauer parenting skills assessment grid (34).

### Infant Development and Risk

The development of the newborn is carried out by means of the Brazelton Rating Scale (BRS), a scale for assessing the behavior of newborns. The aim of this scale is to try to understand the individual characteristics of the newborn by observing his or her strategies for in order to adapt to stimuli in the human and non-human environment. Unlike medical examinations, it focuses on the baby's own activity in participating and responding to the proposed stimuli (35). A “Premi” version of the NBAS exists. Infants being under 1 month of age during hospitalization, current developmental scales [Bayley scales, (36)] are not usable.

### Dyadic Organization

The assessment of the mother-baby relationship is based on interviews and video recordings. The caregiver in charge of the situation meets the dyad alone at least twice daily day. The caretime focuses on the support for childcare (bathing, changing) and the parent-baby relationship, using the PANDA (Postnatal Assessment for newborn development and abilities) grid. This grid, developed by Minjollet et al. (37), focuses on the observation of the newborn in its physiological, behavioral/motor and neurovegetative dimensions. This scale is repeatable and is performed daily by the caregiver. Audio-video recordings of parent/newborn exchanges are also made with the parents' agreement, allowing a detailed observation of early interactions. They can also be used as a support for care (viewing of films by the mothers in the presence of a professional) based on auto-videoscopic techniques.

Because the PANDA beds are an integral part of the Kangaroo Unit, there is daily pediatric and Ob-Gyn care for the aftermath of childbirth throughout the dyad's hospitalization. Immediate follow-ups by specialized physicians therefore take place much more often than a usual stay in a maternity ward would allow (2–3 days for vaginal delivery in France).

### PANDA Post-Hospitalization Follow-Up

At the end of the hospitalization, short-term follow-up is proposed *via* reassessment appointments by the perinatal psychiatrist and the PANDA Unit's carers. Three to six home-visits can also be offered networked with the ambulatory child psychiatry local parent-infant care (see below).

### Infant Psychiatry Follow-Up

Mental health care services: depending on the context, the dyads may be referred to a perinatal follow-up unit which offers care for the child and its family up to the age of 3. A link to adult psychiatric care may also be proposed depending on the evolution of the maternal psychiatric pathology. Educational and social workers are included in Child psychiatric teams and thus are able to provide comprehensive tailored management for both baby and parent.

### Prevention Services: Maternal and Child Protection Service^3^

Maternal and child prevention services: they are essential local players and offer support to families of children under 6. They monitor infant development *via* appointments with professionals and/or support from home helpers and implement public health mandatory measures (such as vaccinations).

### Parental “centers” and Shelters

These centers are publicly funded to take care of mothers, who maybe minors (under 18), and their children up to the age of three. They offer upkeep for the dyad in a community setting or in semi-autonomous flats. The care provided during hospitalization on the PANDA beds makes it possible to target the short-term assistance required for the dyad being cared for. The PANDA Unit carers can then visit mothers and infants in the parenting centers as a “home visit” in addition to ensuring a safety network with parental center staff.

### Child Protection

Child protection services: child protection services can be mobilized from the antenatal period (concerns raised during pregnancy, existing protection measures for siblings). The professionals of the PANDA Unit may also be asked to pass on concerns observed during hospitalization so that a voluntary or mandatory (judiciary) support or protection measure can be put in place at an early stage.

### Panda's Head Start

Six months after official opening, 23 dyads had been admitted to PANDA (see Table 1). Fourteen were included during the antenatal period (61%) and 9 detected during the stay in the maternity ward. On average, women were 27 years old (±7.3, range 17–40; 95%CI 24.1 30.5), and 7 (30%) were under 21 years.

**Table 1:** Age, referral to the unit, primary diagnosis, length of stay and end of stay orientation for the 23 women of the sample.

**Age (years)**	**Admission in the unit**	**Primary diagnosis**	**Length of stay (days)**	**At the end of stay**
17	Before birth	Anxiety disorder	13	Parental center
18	Before birth	Major depressive episode	9	Back to home
18	Before birth	Major depressive episode	14	Parental center
18	Before birth	Personality disorder	28	Out-of-home placement
19	Postpartum	Anxiety disorder	3	Back to home
19	Before birth	Anxiety disorder	22	Parental center
20	Postpartum	Bipolar disorder / Addiction disorder	5	Parental center/Out-of-home placement
22	Before birth	Bipolar disorder	4	Back to home
24	Postpartum	Anxiety disorder	5	Back to home
24	Postpartum	Anxiety disorder	5	Back to home
26	Before birth	Major depressive episode	5	Back to home
26	Before birth	Bipolar disorder	7	Back to home
30	Before birth	Major depressive episode	5	Back to home
32	Postpartum	Addiction disorder	3	Back to home
32	Postpartum	Addiction disorder	8	Out-of-home placement
34	Before birth	Major depressive episode	3	Back to home
34	Postpartum	Anxiety disorder	6	Back to home
34	Postpartum	Major depressive episode	7	Back to home
34	Before birth	Major depressive episode	8	Back to home
34	Before birth	Bipolar disorder	15	Out-of-home placement
36	Before birth	Personality disorder	10	Back to home
37	Before birth	Personality disorder	19	Back to home
40	Postpartum	Major depressive episode	17	Back to home

**Figure 1:**
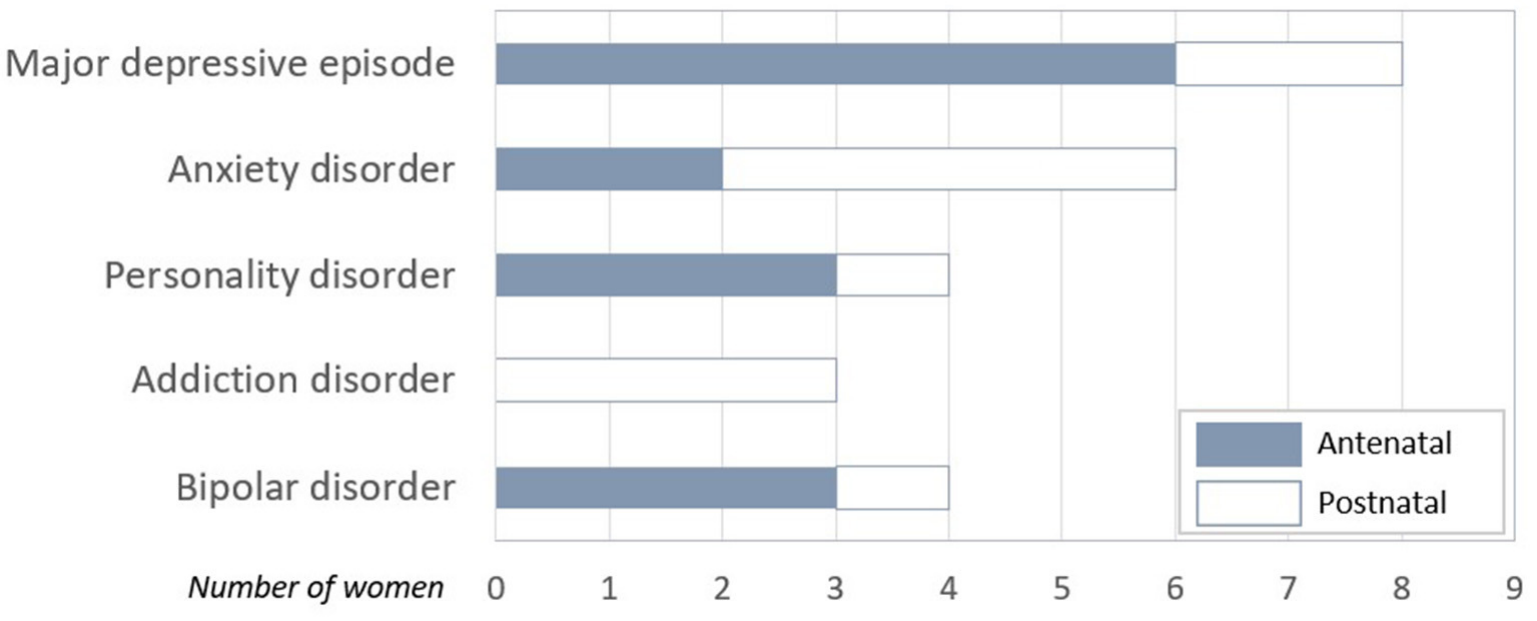
Distribution of PANDA's Women Diagnosis as a function of the moment of inclusion (antenatal/at birth).

The most common primary diagnosis during the antepartum was a major depressive disorder, two-fold that of personality disorder or bipolar disorder alone (see Figure 1). Main diagnosis at birth were general anxiety disorder or addiction disorder.

**Figure 2:**
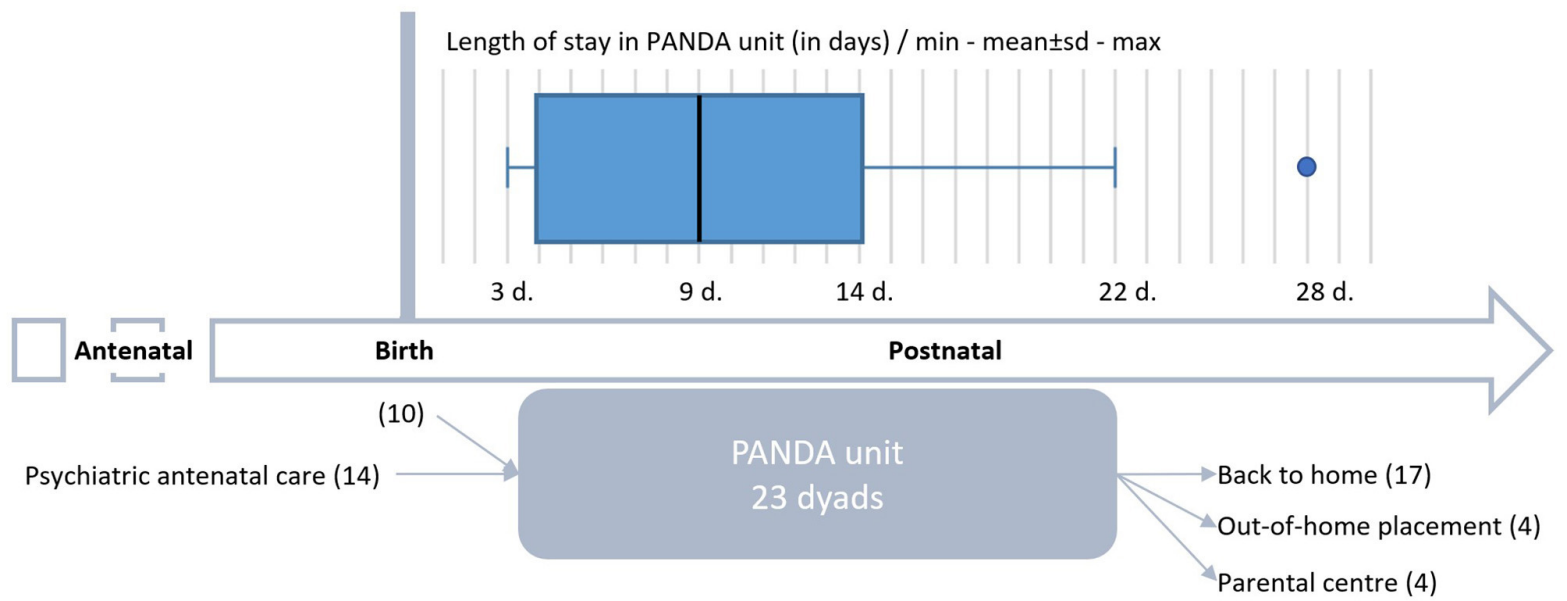
Overview of the PANDA hospitalization course of the first 24 dyads from inclusion to the end of stay.

On average, dyads stayed 9 ± 5.4 days (range 3–22) in PANDA's unit, with one dyad followed up during 28 days (see Figure 2). Half stayed less than 8 days. Women under 21 years tended to stay longer (12 days).

At the end of the PANDA stay, 70% of the women were back in their home with their child (16/23; see Figure 2). Four mothers under 21 and their baby were hosted in a parental center.

An out-of-home placement (OHP) was mandated for 4 babies, two babies of mothers aged under 21 and 2 of mothers aged > 30. One OHP was consecutive to a stay in a parental center, and another OHP was mandated in parallel to the host of the mother in a parental center (baby was placed with mother in the parental home, if mother left home baby would go into foster care).

## Discussion

The PANDA unit is a full-time mother-baby hospitalization facility recently set up within a level 3 maternity hospital. It is an original model for the care of vulnerable dyads as it is located in a pediatric ward. Thus, it provides tripartite care immediately after birth (perinatal psychiatry, neonatology and maternal post-natal care). Most of the referrals are initiated during the antenatal period. Panda was created to respond to situations of high vulnerability in perinatal psychiatry. Our population is on average younger then French mothers nationally (30.3 years), however this does not reach statistical significance.

The Panda unit is somewhat innovative compared to a traditional mother-baby unit by its close links with other general perinatal care professionals, allowing simultaneous comprehensive care (no health without mental health) in the early post-partum period.

### Comprehensive Multidisciplinary Care Nested in Pediatrics

The peripartum is not only a time of higher medical risk for both mothers and their babies, it can heighten complexity of care for dyads or become a window of opportunity. This is partly due to society's view of mental illness during pregnancy and the immediate postpartum, to the manner in which professionals have been trained and to the numerous partners necessarily involved in order to provide comprehensive management. A unit team nested in the general health system, with daily contact and mutual support between professionals is a powerful tool. It provides opportunities for informal and formal interactive training, i.e., shared medical staffs and common continuous medical and carer education. This is a step further to the already positive advantages that have been found in Consultation Liaison teams. Data on how referrals will continue in the long-term will be an essential outcome marker helping to understand how to ameliorate use and organization of Mother-baby units.

### Care at Birth/Continuity

The first visible specific aspect of Panda is that it creates a bridge between antenatal and postpartum care. Most mothers having benefited from perinatal psychiatric care during pregnancy and a Panda unit stay after birth anticipated. This, in itself, means that the same team will ensure that birth does not mean irrevocable change. Mother and neonate do not leave the maternity ward, and the twos and threes care: 2 patients, mother and infant, 3 therapeutic targets, mother, infant and their interactions can start immediately after the birth. Considering the importance of the first weeks and months of life for the establishment of bonding and patterns of parent-infant interaction, this is a unique window of opportunity.

The continuity of care, without any break between the antenatal period and the immediate postnatal period for women is as much “geographic” as it is relational. Indeed, the antenatal and postnatal liaison and ambulatory team in child and perinatal psychiatry team includes PANDA professionals. Thus, the same professionals ensure treatment and care during pregnancy. They can also continue follow-up and or organize within weeks (and not from 1 day to the next) management and care to come when it is deemed to be durable.

The fact that PANDA is located within the neonatology ward ensures that this continuity of care is maintained even in the case of a paediatrically high-risk birth (i.e., small for gestational age, premature birth, risk of withdrawal, etc).

### Very Early Detection of Situations at Risk of Abuse and Neglect

The detection of observations at risk of abuse is another major challenge of care during the peripartum. The issue of care for and prevention of is often a Charybdis and Scylla challenge with very vulnerable dyads. One oscillates between the hope that intensive care and a constructive head start will avoid early abuse and neglect, maintaining the infant on a positive developmental trajectory, thus creating a beneficial developmental cascade; and the risk that too much confidence in this early but short-lived intensive program will unfortunately still not be “good enough” for the infant and therefore insufficient to allow adequate bonding with the infant and a secure relationship for both.

Indeed, observations made during hospitalization can lead to a report being made to child protection services in order to request judiciary protection and mandatory care, thus guaranteeing a secure child protection framework for the infant. Cases where mother and child can be both protected and cared for by the network after hospitalization are for the moment too few to be conclusive. We will need to examine how PANDA unit manages to play both a preventive and therapeutic role by further follow-up in the years to come.

## Limits

First and foremost, 6 months data and a little less than a year since the first dyads were admitted is a too short period of time to draw definite conclusions. The first few months have shown that gathering complete data, on mothers, infants and their interactions, obstetrical and psychiatric, neonatal and developmental, and interactive is in itself a formidable task. Two medical records that need to be both protected and linked are a challenge in itself.

There is a need for information on the interactions and the impact of maternal symptoms on the infant, nevertheless maternal medical confidentiality needs to be protected. In our current judiciary system, both parents have free access to the infant's file notwithstanding the quality of their relationship and the wishes of both parents. Conversely, abstaining from informing the infant's file of parental illness and bonding capacity may hamper adequate assessment and prevent necessary care. These ethical issues are rarely discussed and will need further debate. The use of shared files also requires an adaptation of the electronic documents generally configurated to record medical and biological standardized information rather than verbatim descriptions. They need to be customized to include psychiatric assessment tools (screening and diagnostic for mother and infant) and the interactive tools need to be duplicated automatically in both maternal and infant files when ethically possible, which is far from being the case up until now. We are currently submitting for funding of these essential specific aspects that are necessary if we are to collect data on a larger and more comprehensive scale.

## Perspectives

### Two Clinical Aspects Are in the Starting Blocks

The first is to better organize and systematically allow detection and antenatal care of high-risk situations. This will most probably require an increase in the number of dyads that can be included in Panda care. The first two full time beds were experimental, the initial project required a 6 dyads unit in order to meet the needs of the population of the whole of the Normandy region for which Le Havre is now the Academic tertiary care unit in perinatal Psychiatry.

The second is the follow-up intensive network care needed for this population. A network of day-care and mobile perinatal mother-baby teams need to be progressively implemented on the whole of the Normandy territory in order to propose continuity of tailored care for parents and infants in the months after hospitalization.

A day unit consisting of 3 months once renewable of post-hospitalization care will start current 2022 in Le Havre. It will be a necessary link between the now existing full-time hospitalization and the ambulatory care that is already in place. Presently, in some of the geographical territories of Normandy (as in France) specific outpatient clinics for under-threes are available. They are run by perinatal psychiatrists and sometimes include parental care (38, 39). Intensive day care means more proximity to home. There is a direct need for a general organization and network of care in the region in order to include both rural and urban families inside a large geographical area. Normandy has been pinned as a region with statistically more births of mothers 20 or under as is reflected in our own 23 dyad population. Targeting this specifically vulnerable population is on the table. Both tailored trajectories for young parents and an intensive day-hospital post Panda should start by the end of 2022 (first funding granted).

Finally, research, both clinical and public health oriented is rooted in the project. Panda is an academic (University of Normandy) unit embedded in a tertiary hospital, Groupe Hospitalier du Havre, with an expanding general research unit. The Child and Infant psychiatry department is new and was first implemented at the end of 2017. Clinical externally or self-funded research are an integrated part of its assignments. We know there is a lack of knowledge and prospective monitoring of vulnerable dyads during the perinatal period, most specifically of the quality of interactive exchanges. We are committed to evaluate the difficulties that are specific to each dyad and each of its partners (mother and baby) as well as to assess their evolution at key moments of care. In addition to gaining knowledge of the interactive specificities associated with maternal disorders, we hope that such monitoring will make it possible to consider the benefits and wants of current management (40). We thus hope to further both general and bed-side knowledge with rapid translational gains.

## Data Availability Statement

The original contributions presented in the study are included in the article, further inquiries can be directed to the corresponding author.

## Author Contributions

LV, CH, VB, ED, and GA developed the research protocol, objective hypothesis, and study design. LV, ED, and GA completed the analyses and wrote the first draft of the manuscript. All authors provided constructive feedback on first draft and approved final draft.

## Funding

This study was funded by the Groupe Hospitalier du Havre through l'Agence Regionale de Santé Normandy funding of Pand Unit through the FIOP (Fond d'Innovation et d'Organisation en Psychiatry) in 2020 and the New Measures for Child Psychiatry in 2021.

## Conflict of Interest

The authors declare that the research was conducted in the absence of any commercial or financial relationships that could be construed as a potential conflict of interest.

## Publisher's Note

All claims expressed in this article are solely those of the authors and do not necessarily represent those of their affiliated organizations, or those of the publisher, the editors and the reviewers. Any product that may be evaluated in this article, or claim that may be made by its manufacturer, is not guaranteed or endorsed by the publisher.

## Footnotes

^1^ http://www.marce-francophone.fr/unites-mere-enfant-umb.html

^2^The first 1,000 days refers to a child's life from the moment they are conceived until they have reached 2 years of age (24 months). This is a time when their brain, body and immune system grows and develops significantly.

^3^The maternal and child protection service (PMI) is a departmental service, placed under the authority of the president of the departmental council and responsible for ensuring the health protection of mothers and children.
